# Physical activity and exercise programs for kidney patients: an Italian survey of nephrology centres

**DOI:** 10.1007/s40620-024-01896-w

**Published:** 2024-03-06

**Authors:** Francesca Bulighin, Filippo Aucella, Vincenzo Bellizzi, Adamasco Cupisti, Teresa Faga, Giovanni Gambaro, Giuseppe Regolisti, Alda Storari, Alessandro Capitanini, Yuri Battaglia, On behalf of Working Group of Physical Exercise of Italian Society of Nephrology, Marco Leoni, Massimo Manes, Lorena Traversari, Alessandra Collosi, Paolo Lentini, Antonino Previti, Valentina Fanny Leone, Luigi Vernaglione, Giuseppe Leonardi, Alessandra dalla Gassa, Michele Andreucci, Lorenzo Diliberato, Gemma Patella, Rosa Piperno, Mario Renato Rapanà, Maria Angela Campolo, Luca Piscitani, Giorgio Splendiani, Paolo Ria, Manuela Parrini, Mauro Dugo, Giuseppe Vezzoli, Monique Buskermolen, Gaetano Alfano, Laila Qassim, Maria Amicone, Ersilia Satta, Giancarlo Marinelli, Gabriele Guglielmetti, Carlo Massara, Giuseppe Scaparrotta, Leonardo Calandra, Massimiliano Tosto, Riccardo Maria Fagugli, Federica Baciga, Roberto Scarpioni, Antonio Barilla, Elisa Giglio, Andrea Buscaroli, Francesca Mallamaci, Danio Somenzi, Antonio Favaro, Fulvio Fiorini, Alessandro Naticchia, Sandra Papalini, Veronica Baglio, Sandra La Rosa, Stefano Cenerelli, Marco Amidone, David Micarelli, Marco Pozzato, Fabrizio Valente, Monica Rizzolo, Francesco Bianco, Chiara Caletti, Antonietta Gazo, Paolo Albrizio

**Affiliations:** 1 Department of Medicine, University of Verona, 37129 Verona, VR Italy; 2 Nephrology and Dialysis Unit, Pederzoli Hospital, Via Monte Baldo, 24, 37019 Peschiera del Garda, VR Italy; 3 Nephrology and Dialysis Unit, Casa Sollievo della Sofferenza, 71013 San Giovanni Rotondo, FG Italy; 4 Nephrology and Dialysis Unit, Sant’Anna e San Sebastiano, 81100 Caserta, CE Italy; 5 Department of Clinical and Experimental Medicine, University of Pisa, 56126 Pisa, PI Italy; 6 Nephrology and Dialysis Unit, AOU Mater Domini, 88100 Catanzaro, CZ Italy; 7 Nephrology and Dialysis Unit, AOUI Verona, 37126 Verona, VR Italy; 8 Department of Medicine, University of Parma, 43121 Parma, PR Italy; 9 Nephrology Unit, AUO Ferrara, 44124 Ferrara, Italy; 10 Nephrology and Dialysis Unit, San Jacopo Hospital, 51100 Pistoia, PT Italy

**Keywords:** Exercise training, Haemodialysis, Kidney transplant, Barriers, Research, Inactivity

## Abstract

**Background:**

Data on exercise activities in place, and on the interest for developing them in Nephrology Services in Italy is limited. To address this gap, we carried out this cross-sectional study to investigate the status of physical activity and exercise programs available in Italian Nephrology Centres. Additionally, research priorities on this topic were examined.

**Methods:**

We developed a 14-item electronic survey, which consisted of multiple-choice questions covering exercise training programs, physical assessment, barriers to exercise practice and to exercise programs, exercise and physical activity counselling practices, perceived exercise benefits, literature evidence, and research priorities. Data on the characteristics of the centres were also collected.

**Results:**

Sixty-two responses from Italian nephrology centres were collected. Ninety-three percent of the respondents were aware of the scientific evidence supporting the benefits of regular exercise programs for chronic kidney disease (CKD) patients. Additionally, in 75% of centres the nephrologists believed that physical activity counselling should be performed by the nephrologists. However, only 26% of centres provided exercise programs, mainly for dialysis patients, and 63% never or infrequently assessed physical activity in the context of patient management. Eighty-nine percent of centres reported barriers to implementing exercise programs, including lack of funding, institutional disinterest, patient refusal, and negative attitudes of the healthcare personnel. Forty-six research priorities related to exercise in CKD patients were suggested, with the majority focusing on impact of exercise programs and physical activity on cardiovascular, nutritional, and psychosocial outcomes.

**Conclusion:**

This survey highlights the limited availability of exercise programs and physical activity evaluation in clinical practice in Italian Nephrology Centres. However, the survey also revealed a strong interest for counselling CKD patients on physical activity and implementing exercise prescriptions and interventions.

**Graphical Abstract:**

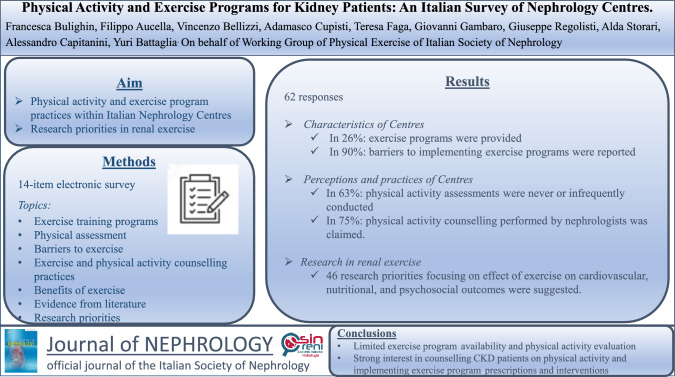

**Supplementary Information:**

The online version contains supplementary material available at 10.1007/s40620-024-01896-w.

## Introduction

Physical inactivity is a common problem in patients with all stages of chronic kidney disease (CKD), reaching its highest prevalence among patients undergoing dialysis and after kidney transplantation [1–3]. Physical inactivity is not only a risk factor for mortality and morbidity in CKD patients, but is also strictly associated with a lower quality of life [4]. Moreover, a decreased level of physical activity is correlated with reduced physical function, muscle mass and muscle strength, as well as an increased risk of frailty, disability, and depression [5]. Conversely, the benefits of physical activity and exercise training in CKD patients is well acknowledged and randomised controlled studies have consistently shown improvement in physical function, cardiorespiratory fitness, muscle strength and quality of life in patients following exercise programs [4].

Consistent with these findings, the 2005 Kidney Disease Outcomes Quality Initiative (KDOQI) clinical practice guidelines emphasised the role of nephrologists and dialysis staff in assessing physical function and counselling CKD patients on physical activity and encouraging increasing physical activity [6, 7]. Subsequently, in the 2012 Kidney Disease: Improving Global Outcomes (KDIGO) clinical practice guidelines, a more detailed recommendation was published, suggesting at least 30 min of physical activity five times a week, based on the patients’ cardiovascular status and tolerance. Recently, the UK Kidney Research Consortium Clinical Study Group's clinical practical guidelines for exercise and lifestyle in CKD patients suggested engaging in 150 min of moderate-intensity aerobic activity per week (or 75 min of vigorous-intensity). Alternatively, a combination of both intensity levels of aerobic activity has been recommended [8]. Nevertheless, in clinical practice, the importance of physical activity is often overlooked, and exercise training programs are underprescribed for CKD patients.

However, adequate physical activity assessment, exercise counselling, and exercise training programs are acknowledged as crucial targets in renal care. To the best of our knowledge, data regarding the extent of exercise activities and interests among Nephrology services in Italy are not available. In order to try to fill this knowledge gap, this cross-sectional study aimed at evaluating counselling and assessment practices regarding physical activity in Italian Nephrology Centres, to identify research priorities on this topic.

## Methods

### Survey development and participants

A panel of experts from the Working Group of Physical Exercise of the Italian Society of Nephrology developed a cross-sectional, questionnaire-based survey. Three Group Members (YB, FA, AC) pre-tested the questionnaire to assess its completion time, ease of understanding, and  relevance of questions. The English version of the questionnaire is available as supplementary material (Supplementary Fig. S1). An electronic version, which could be completed in approximately ten minutes, was developed. The electronic survey was emailed to members of the Italian Society of Nephrology, inviting the directors of each Nephrology Centre or a delegate to fill out only one survey per centre. The description of the purpose of the study and the link to access the secure web-based survey were provided by email. In order to optimize the response rate, two follow-up reminder emails were sent to non-responders. No economic incentive was provided for participating in the survey.

### Survey content

The survey explored the physical exercise practice patterns and resources allocated in Italian Nephrology centres for patients with CKD on haemodialysis (HD) or peritoneal dialysis (PD), and kidney transplant recipients (KTRs). Each survey item was based on a comprehensive literature review and approved by all members of the working group. The questionnaire consisted of 14 items, including questions about exercise training programs, physical evaluation, exercise barriers, counselling practices on exercise and physical activity, benefits and safety of exercise, research priorities, and literature evidence regarding exercise. Additionally, the questionnaire collected data on participants' demographics, practice setting, years in practice of the caregivers, as well as exercise habits. The survey questions were structured with multiple-choice options. Notably, for six questions (specifically 4, 9,11,12,13,14), participants had the option to select more than one response, while for five questions (specifically 5,6,7,8,10), they were asked to choose the type of CKD population (patients in conservative treatment, HD or PD patients, or KTRs). The last question, pertaining to research suggestions, featured an open-text space where respondents could provide their answer. The survey was posted online between March 2022 and December 2022. Ethics committee approval was not required for this study, as it collected the opinions of the physicians, and no patient data were involved.

### Statistical analysis

Data were gathered using a datasheet. Prior to final analysis, an accurate check of data extraction was performed. Only one completed survey per centre was included. Variables were expressed as numbers and frequencies (percentage). SPSS software (version 28, IBM Corp., Armonk, NY, USA) was used for statistical analysis.

## Results

### Characteristics of the centres

**Fig. 1:**
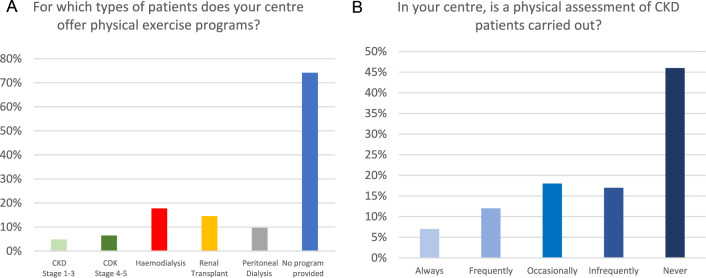
Characteristics of the participating centres

A total of 67 responses were received from 62 Italian nephrology centres; five responses were excluded as duplicates from the same centre. The characteristics of the centres are shown in Fig. 1.

The responding centers were predominantly non-academic hospitals (77%), situated in 45 different cities, distributed across northern (47%), central (19%) and southern (34%) Italy. Additionally, 30% of the centres were in metropolitan areas, and collected large populations. All centres provided care to adult CKD patients, but one that catered to both paediatric and adult CKD patients.

### Barriers

**Fig. 2:**
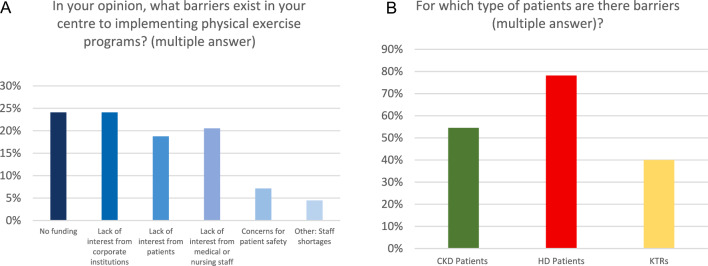
Barriers to implementation of renal exercise programs

Renal exercise programs were available in only 26% of the centres, predominantly for patients on dialysis (52%). Indeed, 89% of the centres reported encountering barriers when attempting to implement exercise programs. The primary obstacles included lack of funding (23%) and of interest by the Local Health Authority (23%), followed by patients’ refusal (18%) and negative attitude of the healthcare personnel (19%) (Fig. 2). These barriers were particularly evident as for providing exercise programs for HD patients, reported in 78% of the centres. Additionally, physical assessment was carried out "always", "frequently" and "occasionally" in merely 37% of the centres. The 6-min-walking-test emerged as the most commonly used tool for physical evaluation, employed in 42% of centres.

### Perceptions and practices of Nephrologists

**Fig. 3:**
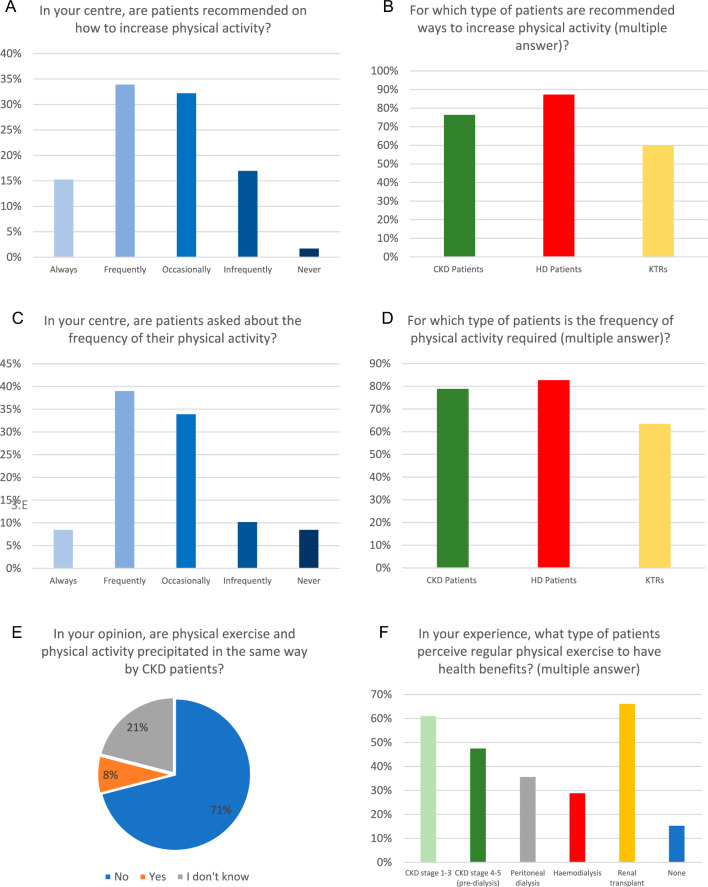
Counselling practice of centres on physical activity

**Fig. 4:**
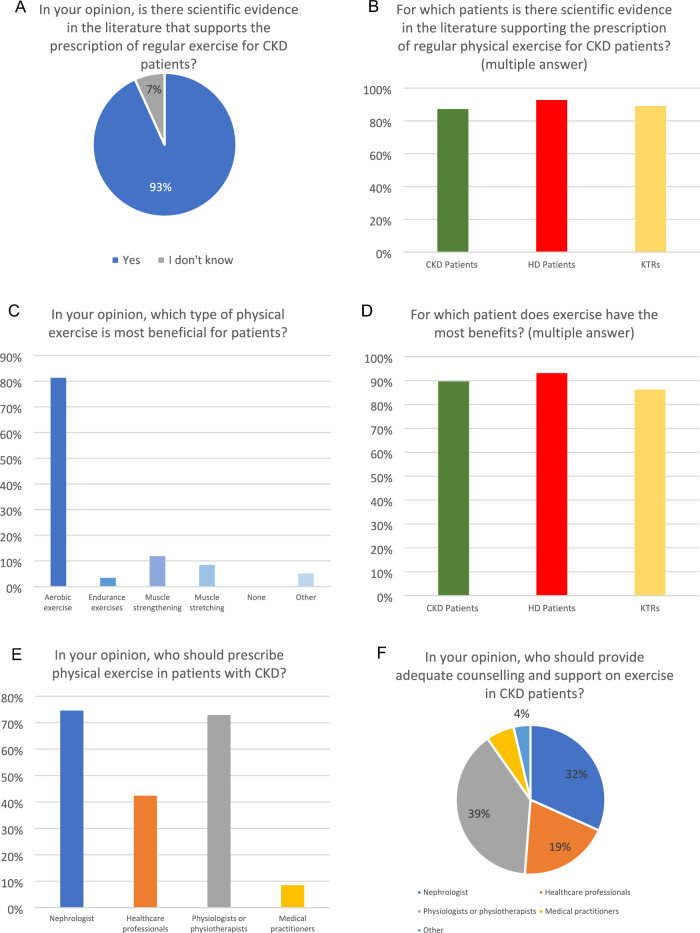
Counselling practice of centres on exercise programs

The exercise programs, physical activity counselling activities, and practice patterns conducted in the centres are illustrated in Figs. 3 and 4. Firstly, 71% of centres agreed that CKD patients were aware of the differences between physical activity and exercise programs. Notably, questions concerning the level of physical activity were frequently (39%) and occasionally (34%) asked to patients with CKD in any stage, in their respective centres. Similarly, advice to increase physical activity was frequently (34%) and occasionally (32%) given, particularly among both HD patients and CKD patients in their centres.

As for kidney transplant recipients and CKD patients in stages I to III, the responders perceived that exercise programs could offer health benefits in 66% and 61% of centres, respectively. In addition, a high level of agreement (93%) was found among the centres regarding the scientific evidence supporting the indication for regular exercise programmes in CKD patients, HD patients and KTRs. More specifically, aerobic exercise was considered the most beneficial training in 81% of centres, while only 12%, 8% and 3% of centres selected muscle strengthening, muscle stretching, and endurance exercises.

### Prescriptions and counselling

Regarding the answer about who should prescribe physical exercise, about 75% of the respondents answered that it should be a prerogative of the nephrologist, followed by 73% who considered that responsibility should be to exercise physiologists or physiotherapists, while 42% believed that other healthcare professionals, including nurses and dietitians, should be involved. Similarly, according to 90% of the responses received, exercise counselling and resources for CKD patients should primarily be provided by exercise physiologists or physiotherapists, followed by nephrologists and healthcare professionals. A small percentage (8-9%) of centres believed that the responsibility for renal exercise programs, counselling and resources should reside with medical practitioners.

### Research in renal exercise

In half of the centres, nephrologists reported that involving their HD patients in an exercise trial was beneficial. However, the healthcare staff in only 21 centres declared their availability in supporting such a study. Conversely, in five centres, the participants did not perceive any interest from CKD patients to be enrolled in a clinical trial on physical exercise.

Concerning the final survey question about the nephrologists' research priorities in the field of physical exercise, it was left unanswered in one quarter of cases. Nevertheless, 46 research priorities related to exercise in a renal context were submitted, and in 80% of cases, two or more topics (totalling 118) were mentioned. The main topics are summarized in Table 1. Specifically, in order to facilitate a systematic organization, topics were arbitrarily categorized into four broad sections, as follows: pathophysiologic mechanisms, exercise prescription, clinical outcomes, instrumental effects. Overall, the most frequently discussed aspect in 90 out of 118 topics was the influence of exercise programs on clinical outcomes, encompassing cardiovascular, nutritional, psychosocial, and CKD-related outcomes. As for clinical outcomes, the question concerning the impact of exercise programs on the patients' quality of life emerged as the most prominent one (14/90). In contrast, the lowest level of interest, approximately 2%, regarded physiopathologic mechanisms and instrumental effects of physical exercise.

**Table 1:** Keywords (number of centers) of identified research topics in renal exercise, in alphabetical order

*Clinical Outcomes*	*Chronic Kidney Disease-Related Outcomes*
All outcomes (1) Comorbidity (2) Hospitalisation rate (2) Mortality (3) Survival (1) Predictive indexes (1)	Chronic Kidney Disease progression (6) Chronic Kidney Disease complications (2) Dialysis efficiency (1) Graft survival (1) Receiving Kidney transplant (1) Start dialysis (1) Uraemic toxins (1) Vascular access (1)
*Cardiovascular Outcomes*	*Pathophysiology mechanisms*
Blood pressure (3) Cardiorespiratory fitness (1) Cardiovascular outcomes (10) Dyslipidaemia (1) Endothelial stress (1) Inflammation (1) Peripheral artery disease (2) Peripheral venous insufficiency (1) Peripheral neuropathy (1)	Acid–base balance (1) Molecular and biological mechanism (1) Myokines (myostatin and irisin), Klotho, FGF23, activin (1) Pharmacokinetics (1) Side effects of drugs (1)
*Nutritional Outcomes*	*Exercise characteristics*
BMI (1) Bone metabolism (2) Frailty (1) Malnutrition (1) Muscle mass (5) Nutritional intake (3) Nutritional parameters (6) Physical benefits (2) Physical function (1) Sarcopenia (1) Weight reduction (2)	Adherence (2) Awareness of disease (1) Costs (1) Feasibility (3) Follow-up (2) Intensity (1) Motivation (1) Prescription (2) Physical function (1) Physical test assessment (1) Risks (1) Safety (1) Socialization (1) Type of exercise (2)
*Psychosocial Outcomes*	*Instrumental Changes*
Depression (1) Psychological effects (4) Quality of life (14)	Bioimpedance (1) Cardiac MRI (1) Doppler peripheral vessels (1)

## Discussion

This is the first survey conducted in Italian Nephrology Centres to assess the exercise programs provided to renal patients, the counselling practices, and perceptions regarding exercise and physical activity, as well as the research priorities in renal exercise.

The most significant finding of this study is the low prevalence (26%) of renal exercise programs provided by Italian centres. These programs were primarily offered to patients on haemodialysis (69%) and to kidney transplant recipients (56%), followed by peritoneal dialysis patients (38%). Notably, only 19% of patients with CKD stages I to III had access to such interventions.

These results are consistent with those of other surveys conducted in various countries. For instance, an international survey among 198 nephrologists practising in Canada, New Zealand, and Australia revealed that 42% of Canadian centres and a striking 81% of Australian centres reported lack of exercise programs [9]. Likewise, a survey carried out by the "Spanish Multidisciplinary Group of Physical Exercise in Kidney Patients" showed that only 19.3% of 264 professionals reported exercise programs for CKD patients in their centres [10].

Furthermore, the evaluation of physical performance, a crucial factor for tailoring personalized exercise programmes, was regularly performed in merely 19% of the Italian centres involved in our study. It is worth noting that this assessment, which does not require sophisticated equipment [11, 12], was not systematically performed even in centres that offered exercise programs.

Nonetheless, it is important to highlight that 93% of centres believed that the scientific evidence was supporting the benefits of regular exercise programs for CKD patients at any stage, as well as for HD patients and KTRs. These data underscore a high level of awareness and knowledge among healthcare personnel in the field of renal exercise, aligning with the recent clinical practice guideline on exercise and lifestyle in 2022, issued by the "UK Kidney Research Consortium Clinical Study Group for Exercise and Lifestyle". This guideline made grade 1C or 1B recommendations, encouraging physical activity and exercise in patients with renal failure, as long as it is not contraindicated [8].

Taken together, our data present a conflicting picture. Among the respondent centres, 74% did not offer any exercise program, and 63% indicated that physical assessment was infrequent or not performed at all. Conversely, only 7% were unaware of the positive impact of exercise programs on the health of their patients. This gap was also described in a previous UK survey by Greenwood et al. [13] in which health professionals acknowledged the fundamental role of exercise in CKD management; however, only 41% of respondents reported the implementation of exercise programmes during dialysis.

One explanation of this discrepancy could be the presence of significant barriers hindering the implementation of the exercise programs, as reported by 89% of the respondent centres. These barriers may be patient-related (e.g. medical factors, psychological factors, or time constraints), healthcare staff-related (e.g. lack of knowledge or time) or healthcare system-related (e.g. insufficient financial resources or institutional disinterest) [14, 15]. Insufficient resources and lack of institutional interest in physical exercise for renal patients emerged as the most frequently cited barriers, accounting for 46% of the multiple choice responses. In order to overcome these barriers, the nephrology community should actively promote awareness of the importance of physical exercise in CKD patients among public institutions and government bodies.

Indeed, the results of a meta-analysis in the general population revealed that physical inactivity was linked to higher healthcare costs in the short-term [16]. Nevertheless, there is a lack of cost analyses specifically involving patients with kidney diseases. In fact, exercise programs may be considered as costly, potentially discouraging the incorporation of exercise as a routine treatment option [17]. To address this gap, it is advisable to plan further studies encompassing cost-effectiveness and cost–benefit to provide insights into the advantages of exercise in renal patients.

Alarmingly, another obstacle we identified was the absence of a proactive attitude towards exercise among healthcare personnel, including medical and nursing staff, in 37% of the enrolled centres. This negative attitude might not only lead to a lack of interest (50% of enrolled centres) in counselling about exercise programs and physical activity, but also result in a reluctance to take responsibility for exercise prescription. Indeed, in 26% of centres the respondents answered that the prescription of exercise programs should be delegated to other professionals. However, these percentages were lower than those reported in a previous Italian survey that focused on HD patients [18], in which 63% of healthcare staff reported that providing advice on exercise was not within the role of physicians or nurses.

Furthermore, our survey shows that although nephrologists in many centres were convinced of  the usefulness of enrolling CKD patients, as well as HD patients, PD patients and KTRs in clinical trials assessing exercise programs, only 41% expressed a willingness to support such clinical trials, thus underlining how active involvement and collaboration of all healthcare professionals is needed in the clinical practice [19].

As for research, our survey showed that 74% of centres proposed challenging research topics, with a primary focus on the impact of exercise programs on cardiovascular, nutritional, and psychosocial outcomes (76% of research priorities). On the other hand, there was little interest (1%) towards studying the pathophysiologic mechanisms of exercise programs. It is worth noting that the most frequently mentioned research priority was the role of exercise in enhancing quality of life. Nevertheless, robust evidence on this topic is currently available among HD patients. For instance, Manfredini et al. conducted a randomised controlled trial in HD patients evaluating the effect of a home-based walking exercise program on changes in QoL, with the Kidney Disease Quality of Life Short Form (KDQOL-SF) questionnaire. The results indicated significant improvement in two components of KDQOL-SF, namely cognitive function score (*p* = 0.04) and quality of social interaction score (*p* = 0.01), at 6 months [20–22]. Additionally, a recent meta-analysis and systematic review highlighted that home-based exercise interventions were significantly associated with improvements in QoL, using the Short Form (36) Health (SF-36) score [23].

The main strength of this survey lies in the fact that participants supplied data concerning exercise programs and counselling practices offered to renal patients within their Nephrology centres.

However, it is important to acknowledge that our survey also has limitations, primarily stemming from the open structure of the study design, including: (1) a positive selection bias, since the participating centres might be the ones that were most sensitive towards, promoting physical activity and exercise programs in renal patients; (2) the participation rate was low (10.1%). Nevertheless, the sampled centres could be considered representative of Italian nephrology practices as the study includes different healthcare settings, including hospitals and dialysis centres, in all Italian regions, encompassing 43% of the main Italian cities.

In conclusion, our survey shows a low prevalence of exercise programs and systematic physical assessment in Italian Nephrology centres. However, the survey discloses a remarkably high level of interest towards recommending physical activity and prescribing aerobic exercise programs for renal patients, along with many logistic barriers.

The nephrology community should make further efforts to reach an agreement on defining the type of exercise to prescribe CKD patients in light of the existing evidence. This can be achieved not only through collaborative initiatives with other professionals, but also by enhancing expertise through workshops, conferences, and courses.

### Supplementary Information

Below is the link to the electronic supplementary material.

Supplementary file1 (DOCX 18 KB)

## Data Availability

The data underlying this article will be shared on reasonable request to the corresponding author.
