# Supplementary material for: Physical activity and exercise programs for kidney patients: an Italian survey of nephrology centres
Source: J Nephrol. 2024 Mar 6;37(3):695–705. doi: 10.1007/s40620-024-01896-w (PMC11150204; doi:10.1007/s40620-024-01896-w)
Supplement: Supplementary file 1 — Supplementary file1 (DOCX 18 KB) [file 40620_2024_1896_MOESM1_ESM.docx]

**Supplementary Materials**

**Supplementary Figure S1.** English version survey

**COUNSELING ON PHYSICAL ACTIVITY IN PATIENTS WITH CHRONIC KIDNEY DISEASE**

Name and Surname:

Gender:

Age:

Years practice:

Setting:

- Hospital
- University-Hospital

Region:

Province:

1. Type of patients under treatment

- Adults
- Pediatric
- Both

1. In your opinion, do patients with Chronic Kidney Disease (CKD) approach physical exercise and physical activity in the same way?

- Yes
- No
- I don't know

1. How often do you engage in physical exercise in your leisure time?

- Almost never
- Less than once a week
- Once a week
- 2/3 times a week
- 4/5 times a week
- Daily

1. For which type of patients does your centre offer physical exercise programs? (multiple choices):

- Patients in pre-dialysis (CKD stage IV-V)
- Patients on peritoneal dialysis
- Patients on hemodialysis
- Kidney transplant recipients
- No programme is offered.

1. In your opinion, what barriers exist in your centre for implementing physical exercise programs? (multiple choices):

- Lack of economic resources
- Lack of interest from local institutional
- Lack of interest from patients
- Lack of interest from medical or nursing staff
- Concerns about the safety of physical exercise
- No barriers
- Other (specify)

If yes, specify patient types (multiple choices):

□ Patients with CKD □ Patients on Dialysis □ Kidney Transplant Recipients

1. In your centre, are patients asked about the frequency of their physical activity?

- Always
- Frequently
- Occasionally
- Rarely
- Never

If yes, specify patient types (multiple choices):

□ Patients with CKD □ Patients on Dialysis □ Kidney Transplant Recipients

1. In your centre, are patients advised on ways to increase physical activity?

- Always
- Frequently
- Occasionally
- Rarely
- Never

If yes, specify patient types (multiple choices):

□ Patients with CKD □ Patients on Dialysis □ Kidney Transplant Recipients

1. In your centre, is a physical assessment conducted for patients with CKD?

- Always
- Frequently
- Occasionally
- Rarely
- Never

If yes, specify the test/s (e.g., SPPB, 6MWT, etc.)

If yes, specify patient types (multiple choices):

□ Patients with CKD □ Patients on Dialysis □ Kidney Transplant Recipients

1. In your experience, which type of patients perceive that regular physical exercise can benefit their health (multiple choices):

- Patients with mild/moderate CKD
- Pre-dialysis patients
- Patients on peritoneal dialysis
- Patients on hemodialysis
- Kidney transplant recipients
- None

1. In your opinion, are there scientific evidence in literature supporting the prescription of regular physical exercise for patients?

- Yes
- No
- I don't know

If yes, specify patient types (multiple choices):

□ Patients with CKD □ Patients on Dialysis □ Kidney Transplant Recipients

1. In your opinion, which type of physical exercise has the greatest benefit for patients with CKD? (multiple choices):

- Aerobic activity
- Resistance exercises
- Muscle strengthening exercises
- Muscle stretching exercises
- None
- Other (specify)

1. In your opinion, who should prescribe physical exercise for patients with CKD? (multiple choices):

- Nephrologist
- Healthcare professionals (nurses, dietitians, etc.)
- Physiotherapists/exercise physiologists
- General Practitioners
- Other (specify)

1. In your opinion, who should provide adequate counselling and assistance on physical exercise for patients with CKD? (multiple choices):

- Nephrologist
- Healthcare professionals (nurses, dietitians, etc.)
- Physiotherapists/exercise physiologists
- General Practitioners
- Other (specify)

1. In your opinion, in which group of patients would it be useful to implement physical exercise through a clinical trial? (multiple choices):

- Patients with mild/moderate CKD
- Pre-dialysis patients
- Patients on peritoneal dialysis
- Patients on hemodialysis
- Kidney transplant recipients
- I don't think my patients would be interested in participating in clinical trials on physical exercise
- I don't know

1. In your opinion, would the staff at your centre be interested in supporting a clinical trial on physical exercise?

- Very interested
- Somewhat interested
- Indifferent
- Not very interested

1. In your opinion, what aspects should scientific research focus on in the field of physical exercise in nephrology?

Free text:
